# Sedentary time of university students before and during the COVID-19 pandemic: Risk groups and pre-pandemic predictors using cross-sectional and longitudinal data

**DOI:** 10.3389/fpubh.2023.1138442

**Published:** 2023-04-05

**Authors:** Sebastian Heller, Kristin Kalo, Antonia M. Werner, Laura Eisenbarth, Jennifer L. Reichel, Lina M. Mülder, Markus Schäfer, Stephan Letzel, Perikles Simon, Thomas Rigotti, Pavel Dietz

**Affiliations:** ^1^Institute of Occupational, Social and Environmental Medicine, University Medical Center of the University of Mainz, Mainz, Germany; ^2^Department of Sports Medicine, Rehabilitation and Disease Prevention, Institute of Sport Science, Johannes Gutenberg University Mainz, Mainz, Germany; ^3^Department of Psychosomatic Medicine and Psychotherapy, University Medical Center of the University of Mainz, Mainz, Germany; ^4^Department of Work, Organizational, and Business Psychology, Institute for Psychology, Johannes Gutenberg University Mainz, Mainz, Germany; ^5^Department of Communication, Johannes Gutenberg University Mainz, Mainz, Germany; ^6^Leibniz Institute for Resilience Research, Mainz, Germany

**Keywords:** epidemiology, health behavior, health promoting setting, higher education, sitting

## Abstract

**Background:**

The present study aimed to (1) assess and compare sedentary time (ST) of university students before and during the COVID-19 pandemic, (2) examine risk groups with regard to ST and the “extent of change” in ST (from before to during the pandemic) in association with sociodemographic (gender, age), study-related (degree aspired to, field of study, semester), and pre-pandemic physical health-related [pre-pandemic physical activity (PA) and ST levels, pre-pandemic BMI class] variables, and (3) investigate whether the change in ST was predicted by these variables.

**Methods:**

Two online surveys were conducted among students at the University of Mainz, Germany—the first in 2019 (before the pandemic) and the second in 2020 (during the pandemic). Participants of both surveys were included in a longitudinal sample. With the longitudinal sample's data, paired *t*-tests, single factor, and mixed analyses of variances were used to examine group differences in ST and the “extent of change” in ST. A linear regression analysis was computed to investigate the influence of the abovementioned sociodemographic, study-related, and pre-pandemic physical health-related variables on the change in ST.

**Results:**

Of the *N* = 4,351 (pre-pandemic) and *N* = 3,066 (in-pandemic) participants of the online surveys, *N* = 443 entered the longitudinal sample. ST increased by 1.4 h/day to critical levels (≥8 h/day) in all subgroups analyzed—even among students who were highly physically active before the pandemic. Students with a low pre-pandemic ST had the largest increase in ST. Pre-pandemic PA level negatively predicted the change in ST.

**Conclusion:**

Even during a global pandemic lockdown, individuals who were previously more physically active and had less ST showed more health-promoting behavior in terms of ST. Therefore, it can be stated that efforts to promote PA and reduce ST are always valuable. Since ST increased and was worryingly high in all subgroups analyzed, all university students should be targeted by multidimensional approaches to tackle ST and promote their health.

## 1. Introduction

On January 7, 2020, Chinese authorities identified the novel severe acute respiratory syndrome coronavirus type 2 (SARS-CoV-2). Due to a rapid increase in cases of the related coronavirus disease 2019 (COVID-19) worldwide, the World Health Organization (WHO) officially declared the spread of COVID-19 to be a pandemic (1).

In response, measures were implemented in March 2020 (in Germany) to limit the spread of the virus, such as the cancellation of mass gatherings, protective mask use in public spaces, and the closure of (public) spaces and facilities, including German universities. Associated with these measures, substantial changes in everyday life, processes, and routines occurred. At universities, the sudden absence of personal contact with peers and faculty; shifting or cancellation of schedules, research, practical work, or exchange programs; profound changes in students' financial and housing situations; and the abrupt switch to online learning (2–4) had far-reaching consequences, not only for students' education but also for their mental health, social behaviors (5), and health-related lifestyle behaviors in general. In the context of health-related lifestyle behaviors, sedentary behavior (SB) appears to be a prominent factor, especially in times of online learning.

The term “sedentary behavior” (SB) is defined as “any waking behavior characterized by an energy expenditure ≤ 1.5 metabolic equivalents (METs) while in a sitting, reclining, or lying posture” (6). In the present paper, we focus on the term “sedentary time” (ST), which refers to the daily time a person engages in any SB. We further created the term “extent of change” in ST, which refers to the difference in ST between specific time points. Studies of the past two decades have shown that populations of high-income countries spend a large amount of their daily awake time sedentary. For example, a representative study among Americans representing the general population showed that they spent 7.7 h/day (or 55% of their awake time) sedentary (7). In a European study with 9,509 participants from England, Portugal, Norway, and Sweden, an average ST of 8.8 h was noted (8). Focusing on the German population, a self-reported median ST of 8.0 h for men and 7.1 h for women has been determined (9). During the COVID-19 pandemic, an increase of 2.1 h in adults' daily ST was reported in a recent meta-analysis of 40 studies (10).

University students are no exception to the general population with respect to ST. In fact, university students appear to be at severe risk of large extents of ST, because the various tasks involved in studying predominantly require large amounts of time spent sedentary (11, 12). For example, according to a meta-analysis that included data from 32 self-report and 8 accelerometer-based studies worldwide, a mean ST of 9.8 h (measured by accelerometer) and 7.3 h (measured by self-report) were reported for university students (11). Additionally, recent studies imply that pandemic-related circumstances also led to significant increases in students' ST: an increase in ST of 52.7% among Spanish (13) students and approximately a doubling of ST (with a high standard deviation) among Italian students (14) were reported using cross-sectional study designs.

From a public health point of view, the abovementioned amounts of ST among university students, and especially the increase in ST during the COVID-19 pandemic, are alarming because SB appears to be a risk factor that is associated with various physiological (15–17) and psychological (18) burdens and diseases, and it may lead to increased mortality (15, 19). Furthermore, according to a meta-analysis by Ekelund et al. (20), the negative effects of SB can only be compensated for with high levels of physical activity (PA)—about 60 to 75 min per day at moderate to vigorous intensity.

Accordingly, it is necessary to reduce and prevent SB. In this context, university students were pointed out as a population of specific relevance, since university students are tomorrow's leaders, decision makers, and parents. Consequently, health promotion and prevention in this collective group would be sustainable and beneficial for the general society (21, 22).

Understanding the conditions and factors associated with ST during the COVID-19 pandemic and the “extent of change” in ST (from before to during the pandemic), as well as the conditions and factors that predicted ST during the pandemic, are necessary for evidence-based planning of health promotion strategies—because the effectiveness of programs is increased when they are adapted to conditions and factors related to ST. Therefore, these potential correlates (factors that are associated) or determinants (factors with a causal relationship) of ST need to be investigated (23). Referring to this, some current research has also examined factors correlating with ST among students during the pandemic, such as gender (13, 24, 25), perceived family affluence (25), and satisfaction with life (24). However, in each of these empirical studies, a cross-sectional design was used, collecting data at a single time point for different dates in the past (before and during the pandemic). In contrast, Romero-Blanco et al. (26) investigated differences in ST in a longitudinal design (before and during the pandemic) within subgroups of alcohol and tobacco consumption, adherence to a Mediterranean diet, motivation, symptoms of anxiety/depression and sociodemographic variables. The researchers concluded that ST increased significantly in all groups except first-year students, overweight or obese students, smokers, and those who did not exercise and did not intend to. However, this study addressed a very specific population; namely, students from the field of health sciences, and intergroup differences were not taken into account. In addition, a previous systematic review that included a small number of seven studies from the United States, Spain, Italy, China, and the United Kingdom summarized that most studies reported a significant increase of ST during the pandemic in undergraduate but not in graduate students (27).

In summary, among the few studies that investigated ST among university students during the COVID-19 pandemic, studies specifically using a longitudinal design and considering differences in ST between subgroups of sociodemographic (age and gender), study-related (semester, degree aspired to, and field of study), and prepandemic physical health-related [pre-pandemic PA level, pre-pandemic ST, and pre-pandemic body mass index (BMI)] variables are lacking. However, longitudinal studies are relevant for examining inter- and intra-individual differences regarding ST. Furthermore, to the best of our knowledge, we are not aware of any study among university students that investigated differences in the “extent of change” in ST, specifically between subgroups of the variables of degree aspired to, field of study, semester, pre-pandemic PA level, pre-pandemic ST, and classes of pre-pandemic BMI. Since SB is seen as a risk factor independently of PA, not only should differences in total ST between groups at one time point and the significance of a potential change in ST over time in a single group be investigated. Rather, differences in the “extent of change” in ST between subgroups are of major importance because the “extent of change” in ST indicates which subgroups experienced a greater change in ST and therefore could feel more strained.

Therefore, the present study aimed (1) to assess and compare ST of university students before and during the COVID-19 pandemic, taking cross-sectional and longitudinal data into account and (2) to examine risk groups with regard to ST and the “extent of change” in ST in association with sociodemographic (age and gender), study-related (degree aspired to, field of study, and semester), and pre-pandemic physical health-related (pre-pandemic PA level, pre-pandemic ST, and pre-pandemic BMI classes) variables. We also aimed (3) to investigate whether these variables predicted the change that occurred in ST from before to during the pandemic.

## 2. Methods

### 2.1. Study design and survey procedure

Two online surveys were conducted among students at the University of Mainz (Germany) as part of an ongoing, evidence-based project on health promotion among students (“Healthy Campus Mainz”). To reduce bias, participation was only possible *via* a link sent by email to all students, using the university's central mail list. Both surveys took place in summer terms—the first in June to August 2019 (before the pandemic; pre-pandemic survey) and the second in June 2020 (during the pandemic; in-pandemic survey). Although both studies were designed as cross-sectional surveys, comparisons of the results for participants of both surveys were enabled within a longitudinal sample, since the majority of items were measured repeatedly, and the respondents created an individualized anonymous code at the beginning of each survey. The survey was performed using the Unipark software, and both surveys followed the same procedure: students were invited to participate *via* email, using the university's central mailing list. The first (pre-pandemic) survey covered questions regarding sociodemographic data, health status, health behavior, and a wide range of potential determinants of health and health behavior. More detailed information regarding the survey procedure and the content of the first survey can be found in Reichel et al. (28). The second (in-pandemic) survey contained additional, more specific questions concerning the COVID-19 pandemic. In an introduction at the beginning of both online questionnaires, the background and purpose of the studies were explained briefly, followed by a statement that participation would be anonymous and voluntary. Informed consent was obtained at the beginning of the survey. Approval to perform the studies was given by the Ethical Committee of the Medical Association of Rhineland-Palatinate (study I: application-number: 2019-14336) and the Institute of Psychology of Johannes Gutenberg University Mainz (study II: No. 2020-JGU-psychEK-S008). The studies were performed in accordance with the Code of Ethics of the World Medical Association (Declaration of Helsinki) for experiments involving humans and the Ethical Principles and Guidelines for the Protection of Human Subjects of Research by the American Psychological Association (APA).

### 2.2. Measures

To determine ST, items 6 and 7 of the short-form International Physical Activity Questionnaire (IPAQ-SF), a valid and reliable instrument, were used (29). To further investigate associations and predictors of ST during the pandemic, besides sociodemographic (age, gender) and study-related variables (semester, degree aspired to, field of study), the following pre-pandemic physical health-related variables were included in the survey: BMI (by computation of body height and body weight) and PA. PA was assessed using the IPAQ-SF.

### 2.3. Data analysis

Descriptive statistics are presented as means with standard deviations (SD) for continuous scaled variables and as numbers and percentages for non-continuous scaled variables.

To analyze differences in ST between the two survey time points of the longitudinal sample, a paired *t*-test was used. To investigate differences between subgroups of sociodemographic, study-related, and physical health-related variables, single factor and mixed analyses of variances (ANOVAs) were used. Therefore, the continuous scaled variables of age, pre-pandemic PA, pre-pandemic ST, and pre-pandemic BMI were categorized. The variable of age was dichotomized at the median. PA was processed according to the IPAQ data processing guidelines (30) and by using a scoring spreadsheet from Cheng et al. (31), and was categorized into levels of weekly PA according to WHO PA recommendations: “physically inactive” for students who did not fulfill minimum WHO PA recommendations [ < 150 min of moderate-to-vigorous PA (MVPA), or < 75 min of vigorous PA (VPA) per week], “moderately physically active” for students who fulfilled minimum WHO PA recommendations (150 to 299.9 min of MVPA, or 75 to 149.9 VPA per week), and “highly physically active” for students who fulfilled minimum WHO PA recommendation for additional health benefits (300 and more minutes of MVPA, or 150 and more minutes of VPA per week) (32). Pre-pandemic ST was dichotomized at a cutoff of 8 h/day. This cutoff was chosen in accordance with the thresholds for mortality that can be found in recent studies (15, 20). BMI was categorized according to the BMI classes given by the WHO (33). Only cases with valid answers regarding ST were included in the analyses. Outliers were excluded from the analysis *via* trimming, using the criterion of ± 2 SD. Additionally, subgroups of < 5 participants were excluded from the analysis.

The mixed ANOVA's interaction effect served as a valuable measure for further examining group differences in the “extent of change” in ST (from before to during the pandemic). To better demonstrate between which specific subgroups potential interaction effects occurred, the variable “extent of change in ST” was computed by subtracting ST of 2019 from ST of 2020. For this variable (“extent of change in ST”), Cohen's *d* was calculated in cases when the previous ANOVA result indicated a significant interaction effect.

Finally, to investigate the influences of the listed independent variables (gender, age, semester, degree aspired to, field of study, pre-pandemic PA level, pre-pandemic ST, pre-pandemic BMI) on the change in ST from before to during the pandemic, a linear regression analysis was computed. The prediction of the change in ST was operationalized by including the autoregressive effect of the variable “pre-pandemic ST—cutoff: 8 h/day” as a predictor. However, only the significant variables of the preceding ANOVAs were included as potential predictors in the regression analysis. Multicollinearity was tested, including the variance inflation factor (VIF). Furthermore, an appropriate sample size for the linear regression analysis was determined by the criterion of 50 events per variable + 100. Bujang et al. (34) revealed that this formula is valid for determining the sample size of observational studies independently of an observed effect size. Accordingly, for example, an analysis with four variables would require a minimum sample size of *n* = 300. Statistical analysis was performed using IBM SPSS Version 27.

## 3. Results

Overall, 4,351 students participated in the 2019 survey (before the pandemic) and 3,066 students participated in the 2020 survey (during the pandemic). Of these, 443 students participated in both surveys, thus building a longitudinal sample. The mean age of the longitudinal sample at baseline (in 2019) was 22.8 years (SD = 3.3 years), and 77.0% (*n* = 341) of the participants were female. Compared to the distribution of age and gender at the University of Mainz as a whole, the mean age was approximately representative (24.7 years was the mean age of the university's whole student body at that time), and women were overrepresented by 18.0 percentage points. With regard to study-related variables, 21.0% (*n* = 91) of the participants were first-year students, 59.8% (*n* = 265) were pursuing a bachelor's degree and 21.0% (*n* = 93) a master's degree, 18.1% (*n* = 80) were aiming for a German state examination (e.g., law and medical students and students of teaching professions), and 1.1% (*n* = 4) were students pursuing different degrees. All sociodemographic and study-related variables of the participants are presented in Table 1.

**Table 1:** Characteristics of the study samples.

	**Longitudinal sample 2019−2020 (*N* = 443)**	**Cross-sectional sample 2019 (pre-pandemic, *N* = 4,351)**	**Cross-sectional sample 2020 (in-pandemic, *N* = 3,066)**
**Gender**, ***n*** **(%)**			
Female	341 (77.0)	3,065 (70.4)	2,225 (72.6)
Male	100 (22.6)	1,246 (28.6)	821 (26.8)
Diverse	2 (0.5)	39 (0.9)	20 (0.7)
**Age, years (mean** ±**SD)**	17–53 (22.8 ± 3.3)	16–73 (23.8 ± 4.4)	16–68 (23.4 ± 4.4)
**Semester (mean** ±**SD)**	1–22 (6.1 ± 3.8)	1–45 (7.1 ± 4.9)	1–35 (6.4 ± 4.5)
**Degree aspired to**, ***n*** **(%)**	(*n* = 443)	(*n* = 4,351)	(*n* = 3,065)
Bachelor	265 (59.8)	2,261 (52.0)	1,709 (55.8)
Master	93 (21.0)	920 (21.1)	645 (21.0)
State examination	80 (18.1)	977 (22.5)	662 (21.6)
Other	4 (1.1)	193 (4.4)	49 (1.6)
**Field of study**, ***n*** **(%)**	(*n* = 442)	(*n* = 4,342)	(*n* = 3,012)
STEM	71 (16.0)	783 (18.0)	506 (16.8)
Social sciences, media and sport	94 (21.2)	774 (17.8)	493 (16.4)
Linguistics, humanities and cultural sciences	87 (19.6)	871 (20.1)	621 (20.6)
Medicine	46 (10.4)	582 (13.4)	341 (11.3)
Law and economics	49 (11.1)	576 (13.3)	479 (15.9)
Teaching	92 (20.8)	665 (15.3)	510 (16.9)
Other	3 (0.7)	91 (2.1)	62 (2.1)

SD, standard deviation; STEM, science, technology, engineering, and mathematics.

Compared to the cross-sectional sample of 2019, the longitudinal sample mainly differs with regard to age-related variables, such as semester and degree aspired to (aside from the variable age itself). The occurrence of this difference is unsurprising, since study graduation increases with student's age over time, and therefore it increases the probability for younger students, and decreases the probability for older students to participate in both studies. At the time of the 2020 survey, the age distribution in the longitudinal sample consequently reflected approximately the age distribution in the cross-sectional samples of 2019 and 2020. There were no considerable differences regarding ST between the cross-sectional samples and the longitudinal sample. However, the overall sample size decreased slightly because not all participants provided valid answers with regard to ST.

### 3.1. Sedentary time of the cross-sectional and longitudinal study samples before and during the COVID-19 pandemic

**Figure 1:**
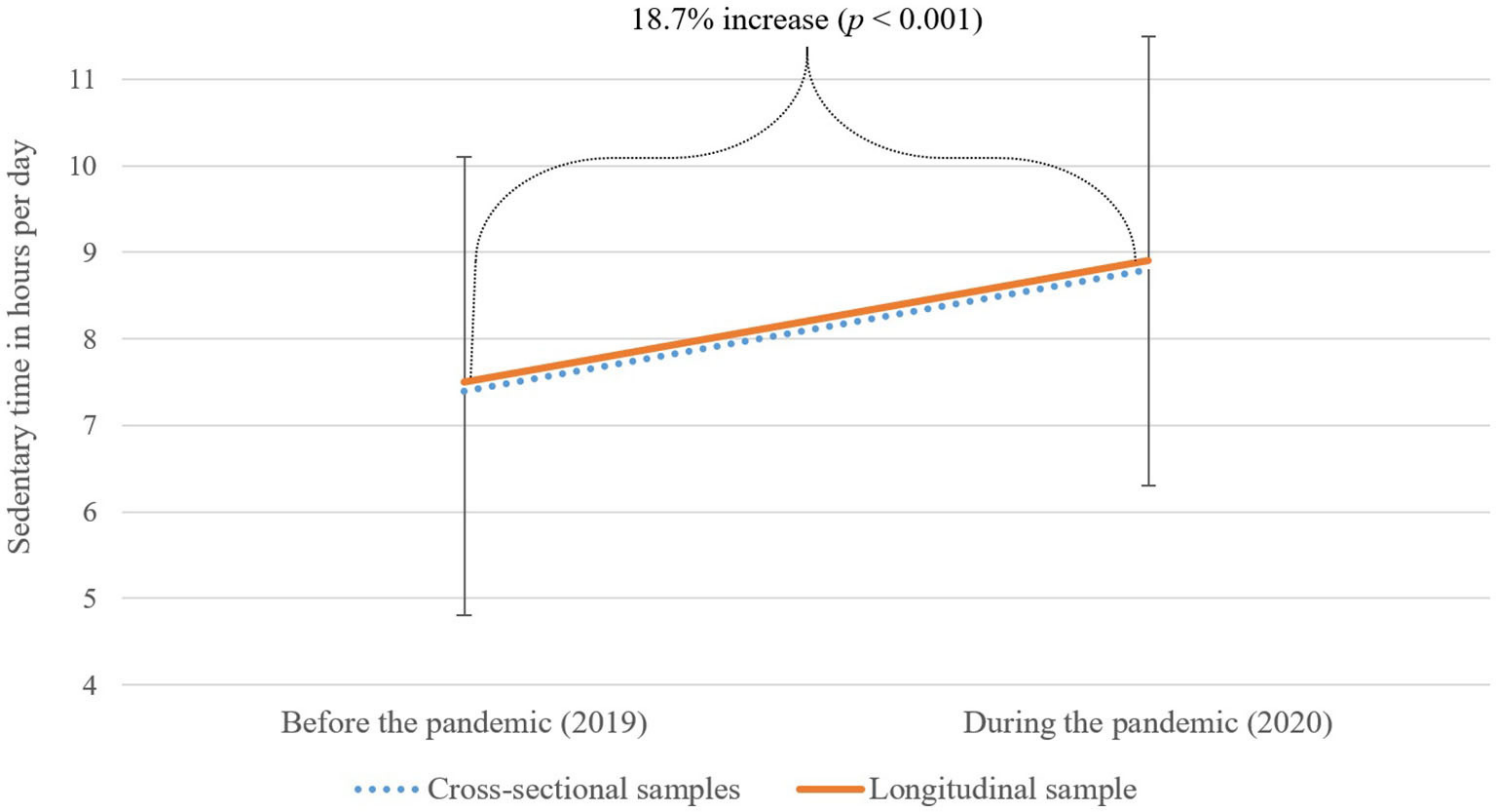
Sedentary time (ST) of the cross-sectional samples (*n* = 3,845 in 2019; *n* = 2,522 in 2020) and the longitudinal study sample (*n* = 378) before and during the COVID-19 pandemic.

Among all participants in the cross-sectional samples, ST was 7.4 (SD = 2.7) h in 2019 (*n* = 3,845; before the pandemic) and 8.8 (SD = 2.5) h in 2020 (*n* = 2,522; during the pandemic). Among the participants of the longitudinal sample with valid ST values (*n* = 378), ST increased significantly by 18.7%, from 7.5 (SD = 2.6) h before to 8.9 (SD = 2.5) h during the pandemic, Cohen's *d* = 2.81, *p* < 0.001, 95% CI [1.14, 1.71] (see Figure 1).

### 3.2. Longitudinal study sample's associations of sedentary time during the pandemic (2020) and of the “extent of change” in ST (2019 to 2020) with subgroups of sociodemographic, study-related and pre-pandemic physical health-related variables

According to our methodological handling of outliers, 52 cases had to be excluded from the analyses: *n* = 47 cases by the criterion of ± 2 SD and *n* = 5 cases by the criterion of subgroups smaller than *n* = 5. Consequently, the sample size for the subgroup analyses was reduced to *n* = 326. Regarding group differences in ST during the pandemic (in 2020), there were no significant differences among the subgroups with regard to gender, age, semester, degree aspired to, and field of study. However, ST in 2020 (during the pandemic) differed between subgroups of pre-pandemic PA levels: ST during the pandemic of students who were highly physically active before the pandemic (8.5 ± 2.1 h/day) was significantly (*p* =0.003) lower compared to those who were physically inactive before the pandemic (9.3 ± 1.9 h/day). Furthermore, there was no significant difference in ST during the pandemic between students who were highly physically active and those who were moderately physically active before the pandemic. In addition, there was no significant difference between students who were moderately physically active and students who were physically inactive before the pandemic.

Regarding the “extent of change” in ST (in hours/day, from before to during the pandemic), no significant differences were investigated between subgroups concerning gender, age, semester, degree aspired to, field of study, or pre-pandemic PA level. This means that in all subgroups of these variables, a comparable increase in ST occurred. In contrast, significant differences in the “extent of change” in ST were revealed between subgroups with regard to pre-pandemic ST (*p* < 0.001) and pre-pandemic BMI classes (*p* = 0.014): The increase in ST was larger among students who had a daily pre-pandemic ST of under 8 h compared to students with pre-pandemic ST above 8 h, Cohen's *d* = 1.08, *p* ≤ 0.05, 95% CI [0.84, 1.31]. Furthermore, compared to students with a pre-pandemic BMI classified as obese, the increase in ST was larger among pre-obese, Cohen's *d* = 0.79, *p* ≤ 0.05, 95% CI [0.18, 1.40], and among underweight students, Cohen's *d* = 0.85, *p* ≤ 0.05, 95% CI [0.15, 1.55].

Further results regarding ST before and during the pandemic, and the “extent of change” in ST can be found in Table 2.

**Table 2:** Means, standard deviations (SD), and analyses of variance in sedentary time (ST) by time point (“2019 pre-pandemic” and “2020 in-pandemic”), sociodemographic, study-related and physical health related variables of the longitudinal study sample.

**Variables and subgroups**	**ST (hours/day) in 2019 (pre-pandemic)**	**ST (hours/day) in 2020 (in-pandemic)**	**Difference in ST between subgroups in 2020**	**Interaction effect between subgroups and time points**	**Extent of change in ST (hours/day) between 2019 and 2020**
	**Mean** ±**SD (*****n*****)**	**Mean** ±**SD (*****n*****)**	**ANOVA**, ***post-hoc*** testing^*^	**F (df)**	**Mean** ±**SD (*****n*****)**
**Gender**					
a) Female	7.2 ± 2.3 (256)	8.8 ± 2.0 (256)	*p* = 0.209	0.05 (1.00, 324.00),	1.6 ± 2.3 (256)
b) Male	6.9 ± 1.7 (70)	8.4 ± 2.2 (70)		*p* = 0.831, $\eta_{\text{p}}^{2}$ < 0.001	1.5 ± 2.4 (70)
**Age**					
a) ≤ 22 years	7.3 ± 2.1 (171)	8.9 ± 2.1 (171)	*p* = 0.130	0.25 (1.00, 324.00),	1.6 ± 2.3 (171)
b) >22 years	7.1 ± 2.3 (155)	8.6 ± 2.1 (155)		*p* = 0.619, $\eta_{\text{p}}^{2}$ = 0.001	1.5 ± 2.4 (155)
**Semester**					
a) First-year	7.4 ± 2.2 (72)	8.6 ± 2.0 (72)	*p* = 0.567	2.90 (1.00, 324.00),	1.2 ± 2.5 (72)
b) Other	7.1 ± 2.2 (254)	8.8 ± 2.1 (254)		*p* = 0.090, $\eta_{\text{p}}^{2}$ = 0.009	1.7 ± 2.3 (254)
**Degree aspired to**					
a) Bachelor	7.3 ± 2.3 (192)	8.9 ± 2.1 (192)	*p* = 0.055	0.40 (2.00, 322.00),	1.6 ± 2.4 (192)
b) Master	6.6 ± 2.0 (66)	8.2 ± 2.1 (66)		*p* = 0.672, $\eta_{\text{p}}^{2}$ = 0.002	1.7 ± 2.2 (66)
c) State examination	7.3 ± 2.2 (67)	8.7 ± 1.8 (67)			1.3 ± 2.4 (67)
**Field of study**					
a) STEM	7.5 ± 2.3 (56)	9.0 ± 2.2 (56)	*p* = 0.328	0.52 (5.00, 319.00),	1.5 ± 2.7 (56)
b) Social sciences, media and sport	6.9 ± 2.2 (68)	8.6 ± 2.2 (68)		*p* = 0.763, $\eta_{\text{p}}^{2}$ = 0.008	1.7 ± 2.1 (68)
c) Philosophy, humanities and cultural sciences	6.8 ± 2.39 (61)	8.4 ± 2.0 (61)			1.6 ± 2.5 (61)
d) Medicine	7.5 ± 2.5 (37)	8.5 ± 1.9 (37)			1.0 ± 2.6 (37)
e) Law and economics	7.4 ± 2.0 (39)	8.9 ± 2.2 (39)			1.5 ± 2.2 (39)
f) Aspiring teachers	7.2 ± 2.0 (64)	8.9 ± 1.9 (64)			1.7 ± 2.1 (64)
**Pre-pandemic PA level**					
a) Physically inactive	7.6 ± 2.0 (72)	9.3 ± 1.9 (72)	*p* = 0.004,	0.46 (2.00, 317.00),	1.7 ± 2.3 (72)
b) Moderately physically active	7.4 ± 2.3 (55)	8.7 ± 1.8 (55)	a–c: *p* = 0.003	*p* = 0.635, $\eta_{\text{p}}^{2}$ = 0.003	1.3 ± 2.4 (55)
c) Highly physically active	6.9 ± 2.2 (193)	8.5 ± 2.1 (193)			1.6 ± 2.3 (193)
**Pre-pandemic ST**					
a) < 8 h/day	5.6 ± 1.4 (174)	8.1 ± 2.0 (174)	*p* < 0.001	89.44 (1.000, 317.00),	2.6 ± 2.0 (174)
b) ≥8 h/day	9.1 ± 1.2 (145)	9.5 ± 1.9 (145)		*p* < 0.001, $\eta_{\text{p}}^{2}$ = 0.220	0.4 ± 2.1 (145)
**Pre-pandemic BMI class**					
a) Underweight	6.7 ± 2.1 (24)	9.0 ± 2.0 (24)	*p* < 0.646	2.60 (3.00, 319.00),	2.3 ± 2.4 (24)
b) Normalweight	7.4 ± 2.2 (223)	8.8 ± 2.1 (223)		*p* = 0.014, $\eta_{\text{p}}^{2}$ = 0.033	1.4 ± 2.3 (223)
c) Pre-Obesity	6.5 ± 2.2 (63)	8.5 ± 2.0 (63)			2.0 ± 2.2 (63)
d) Obesity	8.0 ± 1.4 (13)	8.2 ± 2.6 (13)			0.2 ± 2.6 (13)

^*^Alphabetic characters (e.g., “a–c”) represent significant differences (*p* < 0.05) in ST between respective groups of that variable, computed *via* Games-Howell *post-hoc* testing; $\eta_{\text{p}}^{2}$, partial eta squared; STEM, science, technology, engineering, and mathematics.

### 3.3. The influence of sociodemographic, study-related, and pre-pandemic physical health-related variables on sedentary time during the pandemic among the longitudinal study sample

Since ANOVAs revealed that only pre-pandemic PA levels and pre-pandemic ST were significantly associated with ST during the pandemic, the linear regression analysis was computed with these variables as predictors and ST during the pandemic as the dependent variable. Case processing of the linear regression analysis included *n* = 320 cases, demonstrating appropriate sample size, respectively for events per variable, according to Bujang et al. (34) (50 events per variable + 100, as mentioned in the methods section).

The regression model (see Table 3) revealed that pre-pandemic ST (cutoff: 8 h) positively predicted ST during the pandemic. With the inclusion of this autoregressive effect, the significant negative estimate of pre-pandemic PA level indicates that students who had higher levels of MVPA before the pandemic showed less increase in ST during the pandemic. For descriptive statistics of these predictors, please also see Table 2. The overall linear regression model was statistically significant, χ^2^ (2, 320) = 21.343, *p* ≤ 0.001. Inspecting multicollinearity revealed no collinearity of the chosen variables—with an average VIF of 1.01. The explained variance (Nagelkerke *R*^2^) was 11.3% (see Table 3).

**Table 3:** Significant predictors of the change in sedentary time from before to during the pandemic (2020) in a linear regression analysis.

	***R***^**2**^ = **0.113**	
	**B (SE)**, β	***p***
**Pre-pandemic physical health related variables**		
Pre-pandemic PA level	−0.302 (0.130), −0.123	0.021
Pre-pandemic ST—cutoff: 8 h/day	1.263 (0.216), 0.310	< 0.001

ST, sedentary time; PA, physical activity.

## 4. Discussion

The results of the present study demonstrate that from before to during the COVID-19 pandemic, ST increased to critical levels above the cutoff of 8 h/day in all analyzed subgroups of German university students (mean = 8.9 ± 2.5). The largest increases in ST were found even among students with very low pre-pandemic ST. The results further indicate that pre-pandemic PA level negatively predicted changes in ST between 2019 and 2020.

Referring to the first research aim—namely, to assess and compare ST before and during the pandemic—ST increased significantly by 1.4 h in the longitudinal sample. Between both cross-sectional samples, the difference in ST was 1.4 h, too. Furthermore, STs were closely similar between both the cross-sectional and longitudinal samples, which indicates—when also considering the sample characteristics—a good comparability of the smaller longitudinal sample to the larger cross-sectional samples. The revealed ST during the pandemic is comparable to the ST reported for adults during the pandemic (8.5 h/day) in a recent meta-analysis (10) and approximately in the middle of reported times among the student population (27). Furthermore, the significant increase in ST of 1.4 h/day from before to during the pandemic adds important evidence to the small number of two longitudinal studies on university students' ST. Among these longitudinal studies, the increase in ST varied between 0.7 (UK students) (35) and 2.4 h/day (Spanish students) (26). Moreover, a recent study investigating ST among children and their parents implies that until now, although pandemic-related restrictions have loosened, ST has not returned to levels before the pandemic (36)—a finding that emphasizes the need for appropriate interventions.

With regard to the second research aim; namely, to examine differences in ST and in the “extent of change” in ST among subgroups of sociodemographic, study-related, and pre-pandemic physical health-related variables, some differences occurred, which are discussed hereafter. ST during the pandemic was significantly lower among students who were highly physically active before the pandemic than among students who were physically inactive. Interestingly, there were no significant differences in ST between students who were moderately physically active and those who were physically inactive, and also no differences between students who were highly physically active and those who were only moderately physically active. In other words, ST during the pandemic was significantly different only between the most and the least physically active students. For one thing, the results of significant differences between the most and the least physically active students (regarding WHO PA recommendations) can be put in the context of other pre-pandemic research (37) in which inverse associations between ST and PA were reported—especially with regard to light-intensity PA. Then again, our result that no significant differences between the other subgroups of pre-pandemic PA level were observed verifies the assumption that ST appears as a risk factor mostly independent of moderate- to vigorous-intensity PA, as, for example, demonstrated by Ekelund et al. (20). A possible explanation could be that, due to pandemic-related restrictions, light-intensity PA (e.g., walking to the campus, to the bus station, or between lectures), especially, was impacted the most, which led to an increase in ST, since ST and light-intensity PA are inversely correlated (38).

Therefore, a significantly lower ST was achieved only by students who were highly physically active by engaging in very high amounts of moderate- to vigorous-intensity PA (e.g., by doing home exercise programs). In this context, it should be noted that even the subgroup of students who were highly physically active had a worryingly high ST of 8.6 h during the pandemic, which illustrates the need for measures to reduce ST for all student subgroups. Furthermore, students with a pre-pandemic ST under 8 h still had a significantly lower ST during the pandemic compared to students with a pre-pandemic ST above 8 h. Referring to this, it might not seem surprising that individuals who sat significantly less before the pandemic also sat less during the pandemic. However, it should be noted that exactly this subgroup with a pre-pandemic ST under 8 h had the largest “extent of change” in ST of all subgroups. Therefore, students with a very low pre-pandemic ST should also be observed carefully, as they might feel more physiologically or psychologically strained during the pandemic because they experienced the biggest gain in the risk factor called SB. In addition, even this subgroup with the lowest pre-pandemic ST of approximately 5.6 h sat, on average, more than 8 h during the pandemic. The result that no differences in ST between subgroups of pre-pandemic BMI class were observed is in agreement with the low certainty state of evidence regarding the relationship between BMI and ST (32). However, the significant differences in the “extent of change” in ST between obese and underweight and between obese and pre-obese students could be interpreted in a way that implies that pandemic-related circumstances led to nearly no changes in ST for those who already had high extents of ST before the pandemic—in this case, obese participants. This is in line with the previously discussed results of the subgroup with a pre-pandemic ST above 8 h, who experienced nearly no change in ST (from before to during the pandemic). It is also in line with the findings of Romero-Blanco et al. (26), who stated that those with unhealthy habits (for example, high ST and smoking, in the case of their study) would stick to them and experience no changes during a lockdown. This is also linked with the assumption that grouping healthy and unhealthy factors is habitual in university students: students with a higher ST also have a higher probability of smoking or of having a higher screen time, while exercising regularly is associated with eating more fruit and vegetables and drinking less alcohol. Additionally, in the case of underweight and pre-obese students, they also could have felt at higher risk of a severe case of COVID-19 in the case of infection, and they could therefore have spent even more time on sedentary tasks at home. However, this result must be taken with caution, since there were only *n* = 14 obese participants.

In view of the third research aim, investigating whether the change in ST from before to during the pandemic was predicted by the previously listed variables, pre-pandemic PA level, and pre-pandemic ST were the only significant predictors. This result demonstrates that efforts to promote PA and reduce ST are valuable at all times, since even during events such as a global pandemic lockdown, individuals who were more physically active and had less ST before the pandemic showed more health-promoting behavior in terms of ST.

The present study has several limitations. Unfortunately, it was not possible to examine further associations with PA and BMI during the pandemic, because the respective items were not part of the 2020 survey. This could have been a great enrichment as it would have allowed to interpret ST during the pandemic and the “extent of change” in ST compared to PA and BMI during the pandemic, and their respective “extents of change.” Furthermore, there is also some risk of bias because our study was based on self-reported data. In the case of ST, self-reported data are often linked with an underestimation (39) and could, therefore, especially impact comparisons with smaller subgroups (e.g., pre-pandemic obese students). Based on these previous findings on self-reported (subjective) data vs. objectively measured data in the field of SB and PA, it can be presumed that objective data would show even larger differences between ST before and during the pandemic. In addition, the variable BMI was based on self-reported data, too, and therefore it could have been biased by social desirability: for body height a tendency of overestimation and for body weight a tendency of underestimation has been reported in previous studies (40, 41). Regarding the distribution of the sample, in some ways, the study samples were structurally different from the general student population at the university we investigated. As participation in the study was voluntary, students with a higher interest in health- and disease-related topics might have been more likely to participate than those with a lower interest in these topics. This suggests that our data might have left out a group that could be valuable for disease prevention and health promotion. Nevertheless, our study adds important evidence on university students' ST and its “extent of change” from before to during the pandemic, and therefore, it has the potential to influence the planning of future interventions on SB. Our findings are largely in line with those of previous research showing increased ST since the beginning of the pandemic. However, the longitudinal design of our study, which made it possible to examine inter- and intraindividual differences regarding ST and its “extent of change” with data from before and during the pandemic, has to be emphasized.

## 5. Conclusion with a focus on practical recommendations

Based on a longitudinal design, the present study shows that ST increased to worryingly high levels among all analyzed subgroups of university students. Furthermore, even during a global pandemic lockdown, individuals who were previously more physically active and had less ST showed more health-promoting behavior in terms of ST.

Therefore, it is important to implement concepts targeting all students to reduce SB and promote PA. Current approaches to promoting PA and reducing SB among students usually focus on individual measures. To establish sustainable interventions in the university setting that include both PA and SB, a multidimensional approach with different measures on individual behavioral levels and structural levels is recommended.

One example of a multidimensional approach is the Heidelberg Model of Physically Active Teaching, which aims to improve the quality of education and teaching at universities through measures that promote health and PA (42). In this model, five building blocks are described: (1) Physically activating methods: these give students an opportunity to interrupt sitting and move around, (2) Physically activating courses: courses in which students learn about PA promotion in theory and practice, (3) PA-friendly teaching spaces: PA-friendly design of the teaching and learning environment in the university setting. (4) PA-friendly continuing education: internal university training on physically-activating measures for lecturers in the university setting. (5) PA breaks: interruption of sitting in favor of a short physically active unit within a course.

Taking this approach into account, as part of the Healthy Campus Mainz project, we created an intervention called Health Express during the COVID-19 pandemic. In the Health Express, short videos of 3–5 min are inserted into online lectures (live stream and on-demand lectures). The videos provide content about skills for and methods of promoting health and the management of studies. At the beginning of each video, there is a friendly invitation to stand up and thus interrupt SB. In this way, it combines at least building blocks 2, 4, and 5 of the Heidelberg Model of Physically Active Teaching. Another example is the Smart Moving project (43), in which evidence-based participatory measures, such as (online) active breaks, movement-friendly learning and teaching furniture, as well as a campus route network with nudges for additional movement options, were integrated at a German university.

The application of additional multidimensional approaches combined with further development and research on their effectiveness represent promising possibilities to tackle SB and promote the health of university students in the future.

## Data availability statement

The raw data supporting the conclusions of this article will be made available by the authors, without undue reservation.

## Ethics statement

The studies involving human participants were reviewed and approved by (1) Ethical Committee of the Medical Association of Rhineland-Palatinate and (2) Institute of Psychology of Johannes Gutenberg University Mainz. The patients/participants provided their written informed consent to participate in this study.

## Author contributions

Conceptualization, formal analysis, methodology, and software: SH, KK, AW, and PD. Data curation: SH, KK, AW, LE, JR, LM, MS, SL, PS, TR, and PD. Funding acquisition: SL. Investigation: SH, KK, and PD. Project administration and resources: SL and PD. Supervision: PD. Validation of manuscript content: SH, KK, AW, TR, and PD. Writing—original draft: SH. The manuscript has been read and approved by all authors.
